# T-Cell Receptor Cognate Target Prediction Based on Paired α and β Chain Sequence and Structural CDR Loop Similarities

**DOI:** 10.3389/fimmu.2019.02080

**Published:** 2019-08-28

**Authors:** Esteban Lanzarotti, Paolo Marcatili, Morten Nielsen

**Affiliations:** ^1^Instituto de Investigaciones Biotecnológicas, Universidad Nacional de San Martín, Buenos Aires, Argentina; ^2^Department of Health Technology, Technical University of Denmark, Lyngby, Denmark

**Keywords:** MHC, TCR, CDR, epitope, structure

## Abstract

T-cell receptors (TCR) mediate immune responses recognizing peptides in complex with major histocompatibility complexes (pMHC) displayed on the surface of cells. Resolving the challenge of predicting the cognate pMHC target of a TCR would benefit many applications in the field of immunology, including vaccine design/discovery and the development of immunotherapies. Here, we developed a model for prediction of TCR targets based on similarity to a database of TCRs with known targets. Benchmarking the model on a large set of TCRs with known target, we demonstrated how the predictive performance is increased (i) by focusing on CDRs rather than the full length TCR protein sequences, (ii) by incorporating information from paired α and β chains, and (iii) integrating information for all 6 CDR loops rather than just CDR3. Finally, we show how integration of the structure of CDR loops, as obtained through homology modeling, boosts the predictive power of the model, in particular in situations where no high-similarity TCRs are available for the query. These findings demonstrate that TCRs that bind to the same target also share, to a very high degree, sequence, and structural features. This observation has profound impact for future development of prediction models for TCR-pMHC interactions and for the use of such models for the rational design of T cell based therapies.

## Introduction

A central checkpoint to unleashing a cellular immune response is the recognition of peptides presented by major histocompatibility complexes (pMHCs) by T cell receptors (TCRs). T cells undergo thymal selection. During this selection, T cells with TCRs that either cannot bind pMHCs (negative selection) or bind MHC molecules presenting self-peptides (positive selection) are removed. This process results in a repertoire of T cells with highly specific and selective TCRs, and it is estimated that each TCR can only bind a few thousand (1, 2) distinct pMHC complexes (of a total of more than 20^6^ possibilities, assuming up to 3 MHC anchor positions). TCRs are composed of two subunits: α and β. Each subunit has three loops called complementary determining regions (CDRs) that directly interact with pMHCs. Structural studies from the last 30 years have shown that CDR3 loops usually present the most discriminative interactions with peptides, meanwhile CDR2 loops interact mainly with the MHC and CDR1 loops tend to present soft interactions with both peptide and MHC (3–5). The vast diversity of TCRs allows the recognition of an immense number of different antigens. In the last few years, high-throughput profiling of TCRs have become of routine use and it has been shown that some signatures can be used to describe in general terms the interaction between TCRs and the cognate pMHC complex (6–11). Some studies have demonstrated changes in T-cell populations after several stages of vaccination or exposure to diseases using TCR sequencing (12–16). The specificity of a TCR is most often described using only CDR3 β loop sequences. CDR1 and CDR2 β loops can be included by sequencing TCR β V and J germline regions, thus the full β sequence has also been used to describe the set of TCR signatures (8, 17). Further, the pairing of β with α sequences can be used to allow for more accurate description of the TCR binding specificities (10, 11). This pairing can be obtained through statistical or single cell techniques allowing the most complete modeling of TCR:pMHC restrictions (18–22).

Knowing which pMHC a TCR would bind is a key component toward understanding the mechanisms of T cell immunity. While this can be achieved experimentally, it is an expensive, time-consuming, and low-throughput procedure (23–26). Given this, it would be of great interest to develop means to predict the cognate pMHC target(s) of a TCR based on its sequence alone. At present, however, resolving this task remains a substantial challenge (10, 11, 27). Recently, machine learning approaches have been described (28, 29) that use sequence-based strategies to infer TCR cognate target, but the performance of these methods is severely limited by the very small volume of existing data associating TCRs with their cognate pMHCs target.

In addition to sequence-based methodologies, approaches based on structural information have also been suggested (30–32). As the protein structure often is conserved despite of sequence divergence (33), TCR structure modeling could be helpful to compare binding specificities between TCRs with limited sequence similarity. Some studies have shown how 3D models of the structure of the TCR dimer can be used to complement sequence similarity information and in this way improve our understanding of TCR binding specificities (34–36). Several studies have also achieved promising results in modeling structurally TCR:pMHC complexes and using force field energy functions to assess binding between TCRs and their cognate pMHCs (37–41).

Here, we seek to expand these analyses to further address the issue of TCR similarity and the potential impact on this similarity by the different sequence and structural properties of the TCR and CDR loops. We do this in the context of predicting the cognate pMHC target of a TCR using a simple inference-based approach: for a given TCR query, we search a database of TCRs with known pMHC target(s), rank each entry using a measure of similarity, and finally predict the TCR target based on the most similar pMHC in the database. To develop and benchmark this approach, we define a training set using mouse TCRs binding peptides presented by H-2Db and H-2Kb molecules. Next, the model is applied to an independent evaluation dataset of TCRs that bind peptides presented by HLA-A^*^02:01. We analyze the effect of predicting TCR targets using only CDR3 β loop sequences compared to using both CDR3s, all CDR loops from the β chain and CDR loops from both the α and β chains in the similarity measure. We explore the effect of combining differentially the CDR sequence similarities to boost the prediction performance of our method. Exploiting the fact that full-length paired TCR sequences allow the construction of TCR homology models, we also build TCR dimer structures and predict TCR binding by the means of CDR loops structural similarity. Next, we investigate how such structural information can complement sequence information to improve TCR target prediction, in particular when no reference sequence with high similarity is available for the target annotation.

## Materials and Methods

### Benchmarks for Mouse and Human Alleles

A data set of TCRs with known binding target and peptide MHC restriction to HLA-A^*^02:01, H-2Db, or H-2Kb was obtained from VDJdb (42). Only entries with paired α and β CDR3 loop sequence and corresponding V and J regions annotations were included. Next, to construct full length α and β TCR sequences, V and J sequences were downloaded using their accessions codes from IMGT/GENE-DB (http://www.imgt.org/genedb/) and CDR3 segments extended by aligning the four residues of the C-terminal end of V region to the four N-terminal residues of CDR3 loop and aligning the four residues of the N-terminal end of J region to the four C-terminal residues of CDR3 loop, for both α and β chains. Next, cross-reactive TCRs (the same α and β sequences assigned to bind multiple and distinct pMHCs) were removed. Redundant entries were removed by clustering at threshold of 99% over the average sequence identity between α and β subunits, and selecting the centroid of each group. An overview of the benchmark construction is shown in Table 1 and the number of TCRs for each pMHC is detailed in Table S1. Starting from 3,112 entries, the final benchmark consisted of 984 TCRs binding to H2-Db and H2-Kb, and 520 that bind HLA-A^*^02:01. We used these two datasets for different purposes. The mouse data set was used to develop the best prediction setup, and the human data set was used to evaluate the quality of the model.

**Table 1:** Paired TCRs benchmark statistics.

# Of paired TCRs with known pMHC target	3,112
# Of paired TCRs with known pMHC target excluding cross-reactive TCRs	3,064
# Of paired TCRs binding HLA-A*02:01	831
# Of paired TCRs binding H2-Db	721
# Of paired TCRs binding H2-Kb	999
# Of paired TCRs binding HLA-A*02:01 excluding redundancy	520
# Of paired TCRs binding H2-Db excluding redundancy	466
# Of paired TCRs binding H2-Kb excluding redundancy	482

### TCR Structural Modeling and Loop Detection

The structure of each TCR was modeled using LYRA (35). For each TCR, templates with more than 70% average sequence identity between α and β were included in the blacklist form field of the LYRA server to exclude them from the modeling process. Next, the LYRA output was parsed to detect CDR1, CDR2, and CDR3 loops for both α and β chains.

### TCR Similarity Measures

Three similarity measures were used to identify the cognate pMHC of each TCR: (i) For the global sequence similarity, the sequence identity (SeqID) was calculated separately for the α and β sequences using blast2seq to align the sequences, and was defined for each chain by dividing the number of identical residues by the minimum length between the two aligned chains. (ii) For the CDR sequence similarity, the similarity was calculated by comparing two TCRs using the CDR loops as defined by LYRA annotation. We used the CDR1, CDR2, and CDR3 loops from the α and β subunits. We calculated a similarity between CDRs using the alignment-free Kernel function defined by Shen et al. (43), based on the similarity between all k-mers contained within the sequence of two loops. Briefly, this function is defined as follows: Let B be a BLOSUM62 based similarity measure between two amino acids, as defined by Shen et al. (43) appendix, a similarity between two amino acid sequences *u* and *v* of the same length *k* can be defined as:

$$\begin{array}{l} K\left(u,v\right)=\overset{k}{\underset{i=1}{\prod }}B\left(u_{i},v_{i}\right) \end{array}$$

Based on this, the sequence similarity between two CDR loops *f* and *g* possibly with different lengths as can be defined as:

$$\begin{array}{l} cdr\left(f,g\right)=\underset{\begin{array}{c} u\subset f,v\subset g\\ |u|=|v|=k\\ k=1,\ldots ,\min\left(\left|f|,|g\right|\right) \end{array}}{\sum }K\left(u,v\right) \end{array}$$

Then, we normalized this relation as follows:

$$\begin{array}{l} CDR\left(f,g\right)=\frac{cdr\left(f,g\right)}{\sqrt{cdr\left(f,f\right)cdr\left(g,g\right)}} \end{array}$$

This CDR similarity measure is normalized between 0 and 1 and gives higher values for similar sequences. Finally, (iii) for similarity at structure level, we computed the Root Mean Square Deviation (RMSD) between LYRA detected CDR loops. To do this, Superimposer module of Biopython library was used to structurally aligned all the α and β CDR loops simultaneously using the LYRA numbering scheme to match alpha carbon of the loops. After the alignment, the RMSD between pairs of CDR loops was computed using the following procedure:


      **proc** ComputeRMSD(cdrloop1, cdrloop2):
              RMSD, N = 0, 0
              **for** alpha_carbon1 **in** cdrloop1:
                    alpha_carbon2 =
                          *lookup_nearest_ca*(alpha_
                          carbon1, cdrloop2)
                    alpha_carbon_prime =
                          *lookup_nearest_ca*(alpha_
                          carbon2, cdrloop1)
                    **if** alpha_carbon1 =
                          alpha_carbon_prime:
                               d =
                                   *euclidean_distance*
                                   (alpha_carbon1,
                                   alpha_carbon2)
                               RMSD += d^2^
                               N += 1
              **return** (RMSD/N)^1/2^


### TCR Target Prediction and Pipeline Validation

**Figure 1:**
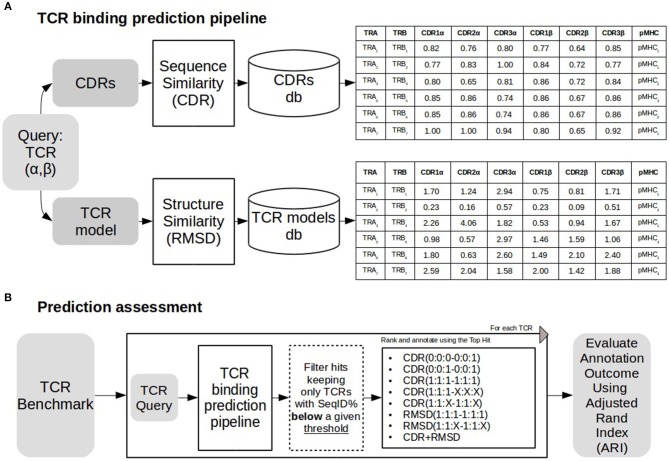
TCR binding prediction and assessment. **(A)** TCR binding prediction pipeline using different similarities. A TCR query is searched against a database of CDRs of different TCRs. Similarities (CDR and RMSD) are calculated as described in methods. Sequence similarity values for each CDR loop are shown in the table above, and RMSD values for structural similarity are shown in the table below. **(B)** The prediction pipeline was assessed annotating each TCR removing TCRs sharing sequence similarity above a define threshold with the query. Both CDR and RMSD similarities were tested with different weights (for details see text). Performance was assessed using Adjusted Rand Index (ARI).

As depicted in Figure 1, a TCR query is defined as a pair α and β chains. The target of a query TCR, is predicted from the most similar TCR in a database of TCRs with known binding targets. Both query and database TCRs were first modeled using Lyra to identify the CDR loops and the structure of the folded TCR. As shown in Figure 1B, we tested the performance of the proposed pipeline in scenarios of varying difficulty when no similar TCRs are available to infer the target of the query. To achieve this, before searching in the database, we removed entries having more SeqID (averaged between α and β chains) with the query than a given cutoff. In order to analyze the performance as a function of the maximum SeqID allowed, we vary this threshold from 70 to 99%. After removal of similar entries, TCRs are ranked with alternative loop weighting schemes with the syntax (1:1:1–1:1:1), where the values in parentheses define the relative weight of each loop. The first triplet identifies the three CDR alpha loops and the second triplet the CDR beta loops. Finally, we assign a pMHC target to the query using the top ranked TCR. We evaluated the pipeline performance at each configuration using Adjusted Rand Index (ARI). ARI is a corrected-by-chance generalization of Matthew's Correlation Coefficient for cases where the data has more than two labels (44, 45). ARI has a value of 1 for perfect predictions, and a value of 0 for the random model. In situations with many labels, the ARI value will often drop substantially below 1, even if a minor subset of predictions is misclassified. Calculations of ARI index were performed using scikit-learn python library.

## Results

In this work, we describe a framework to predict the peptide-MHC (pMHC) binding target of a TCR query based on inference from TCRs with known pMHC binding preference (Figure 1A). A query TCR is scored against a database of TCRs with known binding preference, and the pMHC target is inferred from the top-scoring hit. In a first approach, the scoring is based on sequence similarity over the six CDR loops (for details see methods), and in a second model, structure similarity is added to complement TCR linear sequence information.

To assess the impact of the different loops on the predictive power of the model, a series of different weighting schemes were evaluated (Figure 1B). In the simplest scheme, only the CDR3 β loop was included in the model [i.e., weighting scheme (0:0:0–0:0:1)]. Secondly, we included the full β sequence by adding the CDR1 and CDR2 β loops with weights (0:0:0–1:1:1). In the third model, both α and β subunits were included using either an equal weighting scheme (1:1:1–1:1:1), a scheme with increased CDR3 loops relative weight [(1:1:2–1:1:2) or (1:1:4–1:1:4)], or a scheme with differential weighting between β and α subunits [(1:1:1–2:2:2) or (1:1:1–4:4:4)]. In the case of the global sequence similarity (see methods), a weighting scheme combining α and β subunits was used where SeqID(0:1) stands for using only β subunit, SeqID(1:1) for using both α and β subunits and SeqID(1:2) for doubling the β weight over α.

**Figure 2:**
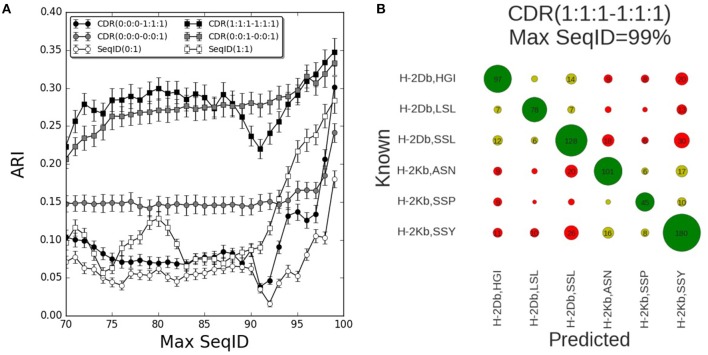
Prediction performance for H-2 (mouse) benchmark. **(A)** TCR binding prediction performance as a function of Max SeqID for different similarities and weighting schemes. Increasing CDR similarities from using only CDR3 β, CDR(0:0:0–0:0:1) to all α and β CDR loops, CDR(1:1:1–1:1:1). When we use only β chain we filter sequences using SeqID(0:1) and when we predict using both chains we filter sequences using SeqID(1:1). Error bars are estimated using bootstrap with 1,000 iterations on the final prediction outcome. **(B)** Confusion matrix of the prediction outcome used for ARI calculation. Predictions performed using the model with equal weights for each CDR loop [model CDR(1:1:1–1:1:1)] using a Max SeqID TCR similarity threshold of 99% have an ARI value of 0.35. Rows and columns are labeled with the MHC mouse allele and the first three letters of the peptide. Green circles are correctly predicted TCRs, light green circles represent correctly predicted MHC but wrong peptide, and red circles are for wrong MHCs. Numbers <5 are omitted for clarity.

The results of benchmarking these different models on the mouse benchmark data set are shown in Figure 2A. Here, the performance measured in terms of the Adjusted Rand Index (ARI) of each model is shown as a function of the maximum sequence identity (Max SeqID) allowed between the query TCR and TCR database (for details see methods). An example of this is given in Figure 2B. Here, the confusion matrix underlying the calculation of ARI is shown for the model CDR(1:1:1–1:1:1) in the situation allowing Max SeqID of 99% corresponding to the extreme right point in the performance curve. The corresponding ARI value is 0.35 and the accuracy 66%.

The performance of each model was tested for a range of maximum sequence identity allowed between the query TCR and TCR database (Max SeqID%) from 70 to 99%. As shown in Figure S1, the minimum SeqID% for each TCR to other TCRs binding the same pMHC is below 32%, which means that even if we filter out TCRs that share more than 70% SeqID when we search the TCR database, we will always, for each query, find at least one other TCR sharing the given target. Predicting the correct cognate target should therefore be possible in all cases. Additionally, we evaluated the performance of a random model, assigning a random TCR in the database search and obtained, as expected, an ARI value close to zero for all Max SeqID thresholds (Figure S2).

First, we investigated how the predictive performance of the framework was improved as the sequence information included in the model was increased. The prediction model defined by only including the CDR3 loop of the β chain [model CDR(0:0:0–0:0:1)] had improved performance compared to the model using SeqID with the whole β sequence [SeqID(0:1)]. Adding the CDR1 and CDR2 loops from β subunit to the model [CDR(0:0:0–1:1:1)] led to a general drop in performance compared to using the CDR3 alone (Figure 2A). Only for very high similarities (Max SeqID>97%) the performance improved when adding these loops in addition to CDR3, suggesting that incorporation of CDR1 and CDR2 loop similarities might be detrimental to the model. This is further illustrated in Figure S3, where we show the confusion matrices for the two models model CDR(0:0:0–0:0:1) and CDR(0:0:0–1:1:1) evaluated at a Max SeqID threshold of 92%. This figure clearly demonstrates that the fraction of cases with wrongly predicted MHC target is increased for the model including the CDR1 and CDR2 loop information.

Next, we added the paired α sequences to the model. Using the complete α and β sequences [model SeqID(1:1)] led to an improved performance compared to using only the β sequences [model SeqID(0:1)]. Likewise, the model using the α and β CDR3 loops together (model CDR(0:0:1–0:0:1) outperformed the model including only CDR3 β model [CDR(0:0:0–0:0:1)]. This model also outperformed the model including the two full length sequences [model SeqID(1:1)]. When including the CDR1 and CDR2 from both α and β subunits using a (1:1:1–1:1:1) weighting scheme, we observed a general improvement of performance compared to using only the paired CDR3 loop sequences, but also here, we observe a small drop in performance around a Max SeqID of 91% suggesting that a differential weighting would be needed over the CDR3 loop similarity.

Up to this point, we have analyzed the predictive performance as a function of maximum SeqID% allowed between the query TCR and any entry in the TCR database. This approach could clearly be unfair to models based on full length sequence identity such as SeqID(1:1), since we exclude possible database entries based on the same measure used to define the best database target. To assess to what degree this is the case, we assessed the prediction outcome also as a function of CDR3 similarity, incrementally including more similar CDR3 α and β loops while predicting using different weights (Figure S4). This benchmark confirmed the earlier conclusions that model CDR(1:1:1–1:1:1) outperformed all other models including SeqID(1:1).

### Adjusting Weights to CDR Loop Similarity

**Figure 3:**
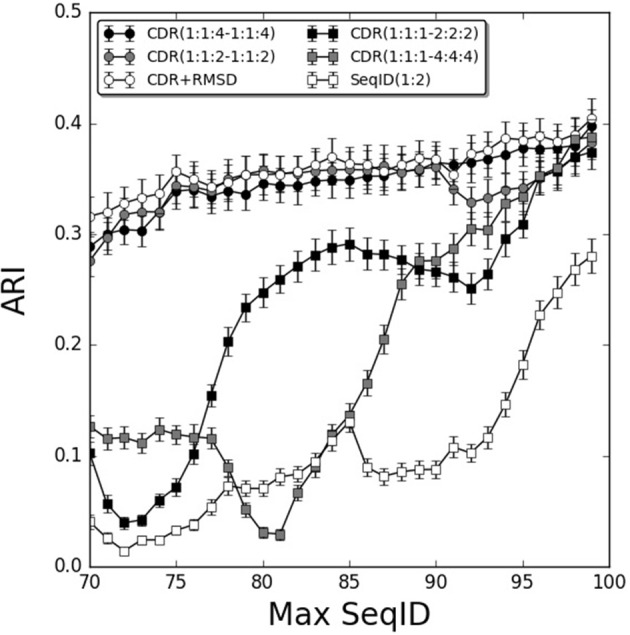
Improving weighting schemes and adding structural similarity. Prediction performance as a function of Max SeqID using different weights for CDR loop similarities. Adding structural similarity CDR+RMSD with W = 0.9. Error bars are estimated using bootstrap with 1,000 iterations on the final prediction outcome.

To further investigate the relative contribution of each CDR loop, we investigated differential weighting schemes for CDR3 over CDR1 and CDR2 loops (Figure 3). The schemes are defined using a (1:1:X−1:1:X) scheme varying the relative weight on the CDR3 loop or a (1:1:1–X:X:X) scheme varying the relative weight of the β over the α chain.

We found improvements in the prediction when different weights were applied to the CDR3 loop, and the optimal performance was found for the model CDR(1:1:4–1:1:4). This model outperformed both the flat model [CDR(1:1:1–1:1:1)], the model with double relative weight on CDR [CDR(1:1:2–1:1:2)], and demonstrated a monotonic increased in performance from low to high sequence identities. Moreover, doubling and quadrupling the β subunit weight over the α subunit was investigated [models CDR(1:1:1–2:2:2) and CDR(1:1:1–4:4:4)] but these weighting schemes consistently led to decreased predictive power compared to the flat model [CDR(1:1:1–1:1:1)]. Other weighting schemes were investigated but did not lead to consistent improvements in the prediction accuracy (data not shown).

### Adding Structural Modeling Improves TCR Cognate Target Prediction

We next extended the models to also include structural information. We constructed TCR models using LYRA with templates sharing no more than 70% SeqID with the target to avoid the effect of overfitting in the modeling process. Then, we calculated CDR loops structural similarity by computing the RMSD between two given TCRs and used these loops similarities to predict each query (for details see Figure 1 and methods). By itself, the structure-based model performed worse than the sequence-based approach described above (Figure S5A). Furthermore, the flat model RMSD(1:1:1–1:1:1) outperformed the model RMSD(1:1:4–1:1:4) with differential CDR loop weighting (Figure S5A). This observation is most likely due to the fact that CDR3 loops in general are modeled with relative low accuracy, as shown previously by Gowthaman et al. (36), limiting the predictive signal contained within the structure of these loops. Finally, we screened relative weights for combining structural and sequence information in a single model. We integrated sequence and structural similarities with a weight W in the linear model defined below:

$$\begin{array}{l} CDR+RMSD=W^{*}\left[1-CDR\left(1:1:4-1:1:4\right)\right]\\ \;+{\left(1-W\right)}^{*}RMSD\left(1:1:1-1:1:1\right)/5.0 \end{array}$$

Screening different values of W, the optimal performance was W = 0.9 (Figure S5B). The performance of this combined model was only slightly better than the best sequence based model CDR(1:1:4–1:1:4), with a gain more pronounced for lower values of Max SeqID (Figure 3). We assessed the significance of this performance gain using bootstrapping, and we found the gain to be statistically significant only at SeqID = 70% (Figure S6).

### Independent Model Evaluation on Human TCR Targets

**Figure 4:**
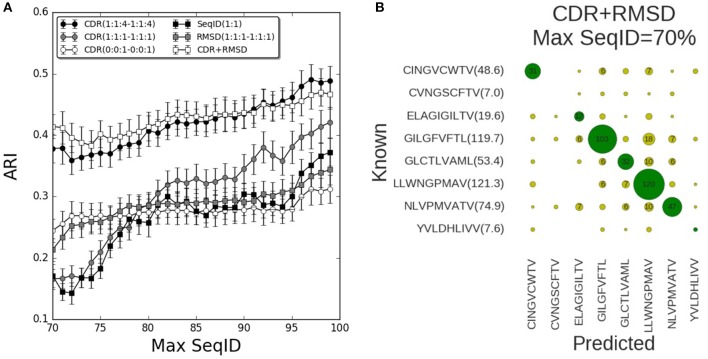
Validation of pipeline performance on HLA (human) benchmark. **(A)** Prediction performance as a function of maximum sequence identity for different similarity models and relative weighting schemes. The combined model CDR+RMSD integrating structural similarities was made using W = 0.9 (see text). Error bars are estimated using bootstrap with 1,000 iterations on the final prediction outcome. **(B)** Confusion matrix for the CDR+RMSD model at a Max SeqID threshold of 70% (with an ARI of 0.41). Green circles are correctly predicted peptides and yellow circles represent wrong peptide predictions. Numbers lower than 5 are omitted for clarity. In parentheses is displayed the average number of TCRs that bind the same peptide and remain after removing entries with Max SeqID > 70%.

We now turned to the HLA-A^*^02:01 data sets to validate the prediction pipeline and the conclusions obtained from the mouse data. As also observed in the mouse benchmark, the performance using SeqID(1:1) was lower than using CDR similarities (Figure 4). Consistently, the differential weighting scheme (1:1:4–1:1:4) resulted in better predictions compared to using the (0:0:1–0:0:1) and (1:1:1–1:1:1) schemes. We assessed the CDR+RMSD model combining sequence and structural information using the relative weight W = 0.9 optimized on the mouse data, and found a significantly (*p* < 0.04, bootstrap test) improved performance for Max SeqID <72% compared to the CDR(1:1:4–1:1:4) model (Figure S6). For Max SeqID in the range 75% < SeqID <90%, model CDR+RMSD slightly outperformed the sequence based CDR(1:1:4–1:1:4) model, but this difference was not statistically significant (*p* = 0.4, bootstrap test). As expected, the addition of structural information at higher value of Max SeqIDs (Max SeqID>90%), did not improve the predictive power of the model.

As a final remark, we investigated the distribution of prediction accuracy for each peptide at Max SeqID = 70% for the combined CDR+RMSD model (Figure 4B). It is apparent that the prediction quality varies substantially between peptides. This variation is, to a very high degree, related to the number of TCRs sharing the given peptide target. For instance, the model performs rather poorly for the peptides CVNGSCFTV and YVLDHLIVV, both characterized by a very small number of TCRs sharing them as target. The CINGVCWTV, ELAGIGILTV, GLCTLVAML, and NLVPMVATV entries all share 20 or more TCR entries and the model obtained accuracy values between 40 and 60%. Consistently, for the most populated cases GILGFVFTL and LLWNGPMAV with more than 100 TCRs sharing each peptide, the model obtained an accuracy of 72% (103/144) and 85% (120/142), respectively. These observations underline, as expected, the very high dependency of the accuracy of the proposed modeling framework to the number of TCRs in the database known to bind a given peptide. It also suggests that increasingly accurate predictions will be achievable as the space of pMHC-TCR sequences becomes populated by new experimental data documenting these interactions.

## Discussion

The activation of T cells depends on specific interactions between TCRs recognizing peptides presented by MHC. These interactions depend almost exclusively on CDR loops. Generally, analyses of T cell repertoires have been oriented to TCR β chains because obtaining the paired α sequence is more difficult and costly. Further, clonal expansion is often analyzed by the means of sequencing only the CDR3 loop of the TCR β sequence (11, 33). While these constrains on the TCR sequence being generated and analyzed might be justifiable seen from a cost perspective, it is clear that focusing only on the TCR β chain, and in most cases only of the CDR3 β loop potentially has large and limiting implications for the conclusions drawn and information harvested from such TCR sequence data.

We found the predictive power of the model to improve substantially when including the α in addition to the β chain. We also showed that, as expected, focusing on CDR loops rather than the full-length protein sequence led to improved performance. Investigating the relative importance of the different CDR loops for the predictive power of the model, we found an increased performance for models with higher relative weight on the CDR3 loops compared to CDR1 and CDR2. Finally, we demonstrated that the inclusion of structural similarities in the model improved, modestly but consistently, the accuracy of the target prediction, in particular in situations where no sequence with high similarity is available in the TCR database. While being statistically significant, gain in predictive performance obtained by including structural information was limited. We expect this to change, as the accuracy of TCR structural modeling tools improve (in particular for the two CDR3 loops) and the number of available TCR structures (to be used as templates) increases. However, as data available is limited in terms of the diversity and the number different epitopes involved, we find it impossible to draw conclusions about how these interactions mediate different T cell responses. Also, we neither have enough data to tackle the importance of each loop in the recognition of different MHC alleles as we only have enough information about HLA-A^*^02:01 for human, and H2-Kb and H2-Db for mouse. As well, we have only MHC class I data, and it would be of great importance to have more MHC class II binding TCRs to get better insights on the difference between CD4 and CD8 T cell interactions with antigens. We hope some day would be more data and more diverse in all of these aspects in order to learn more about the regulation of the immune response.

Predicting TCR cognate targets is a very difficult challenge and the main limit is imposed by the lack of data availability on this huge sequence space. This puts some barriers in our understanding of TCR binding specificities and, the issue gets even more complicated if we try to predict unknown binding specificities. If this problem would be solved, our capability to predict T cell responses would be dramatically improved, but we are still far from achieving it. In the present work, we present a framework to predict specificities to known cognate targets of TCRs using an inference-based model, seeking to understand the importance of using paired TCR sequences.

Despite the very simple modeling approach taken here, these findings clearly demonstrate both that paired full length sequence information is essential for the accurate assessment of TCR function, and that given such information, simple structural, and sequential properties that are common between TCRs that share cognate binding target can be identified. This observation not only underlines the need for the generation of large TCR data sets containing the full information about the triad involved in the TCR:pMHC synapse, using for instance single cell based methods (46), but also suggests that prediction of TCR:pMHC interactions is feasible and thus lays the foundation for the development and application of such models to rational design of T cell based therapies.

It is however critical to stress that due to the availability of data the work and results presented here are limited to the MHC class I and CD8 TCR system. While MHC class II and CD4 TCRs share large structural and functional similarities to this system, several important properties sets them apart—in particular imposed by the longer peptide resituating in the MHC class II binding cleft. Likewise, are the analyses presented limited to cover only three different MHC class I molecules, and certain caution should be taken when extrapolating the conclusions to all class I molecules. However, as more data become available, the framework proposed here can readily be applied to investigate if the presented conclusions are indeed applicable to the general TCR-pMHC system.

Finally, it is essential to reiterate that we here have presented a framework to predict cognate targets of TCRs using an inference-based model, seeking to understand the importance of using paired TCR sequence and structural information. Using an inference-based model imposes very large limitations on the applicability of framework for the task of general prediction of the cognate target of TCRs since it depends on the availability of other TCRs sharing the same target, and hence does not allow for true ab initio predictions.

This said, our findings demonstrating an improved predictive power when including information from the α chain in addition to the β chain hold consistently true throughout our benchmark calculation. This important observation not only underlines the need for the generation of large TCR data sets containing the full information about the triad involved in the TCR:pMHC synapse, using for instance single cell based methods (46), but also demonstrates that TCRs with a common cognate target share tractable common sequence and structural properties suggesting that prediction of TCR:pMHC interactions is feasible and thus lays the foundation for the development and application of such models to rational design of T cell based therapies.

## Data Availability

The datasets for this manuscript are not publicly available because they are already public access data. Requests to access the datasets should be directed to Esteban Lanzarotti, elanzarotti@dc.uba.ar.

## Author Contributions

EL performed analysis and drafted the paper. PM and MN participated in the design. MN wrote the final version of the manuscript.

### Conflict of Interest Statement

The authors declare that the research was conducted in the absence of any commercial or financial relationships that could be construed as a potential conflict of interest.

## Supplementary Material

Figure S1 Minimum inner SeqID similarities between the TCRs that bind the same target. **(A)** Mouse alleles: H-2Kb, H-2Db. **(B)** Human allele HLA-A^*^02:01.

Figure S2 Prediction pipeline random performance. Random prediction is performed picking a random TCR when we search the database for the TopHit. Error bars are estimated using bootstrap with 1,000 iterations on the final prediction outcome.

Figure S3 Contingency matrix for the prediction of mouse pMHC binders at a 92% Max SeqID threshold. **(A)** Prediction performed using only CDR3 β loop [model CDR(0:0:0–0:0:1)] with an Adjusted Rand Index (ARI) equal to 0.14. **(B)** Prediction performed using CDR1, CDR2, and CDR3 loops weighted equally [model CDR(0:0:0–1:1:1)] with an ARI = 0.04.

Figure S4 TCR prediction performance as a function of maximum CDR3 similarities. Using increasing CDR similarities from using only CDR3 β, CDR(0:0:0–0:0:1) to all α and β CDR loops, CDR(1:1:1–1:1:1). When we use only β chain we filter sequences using CDR(0:0:0–0:0:1) and when we predict using both chains we filter sequences using CDR(0:0:1–0:0:1). Error bars are estimated using bootstrap with 1,000 iterations on the final prediction outcome.

Figure S5 Looking for the best weights to combine structural similarity using the mouse benchmark. **(A)** RMSD prediction performance as a function of maximum SeqID allowed in the database for different weights. **(B)** Grid search for combined model weight between sequence and structural similarities different SeqID% cutoffs.

Figure S6 Bootstraping *p*-values comparing CDR+RMSD against CDR(1:1:4–1:1:4) as a function of maximum SeqID%. Tests were performed with 1000 iterations bootstrapping on the final prediction outcome for both models and *p*-value is obtained dividing by 1,000 the number of times the ARI value of CDR(1:1:4–1:1:4) resulted better than CDR+RMSD.

Table S1 Number of TCRs in datasets used for model discovery (mouse) and validation (human).
