# Supplementary figures and images for: T-Cell Receptor Cognate Target Prediction Based on Paired α and β Chain Sequence and Structural CDR Loop Similarities
Source: Front Immunol. 2019 Aug 28;10:2080. doi: 10.3389/fimmu.2019.02080 (PMC6724566; doi:10.3389/fimmu.2019.02080)

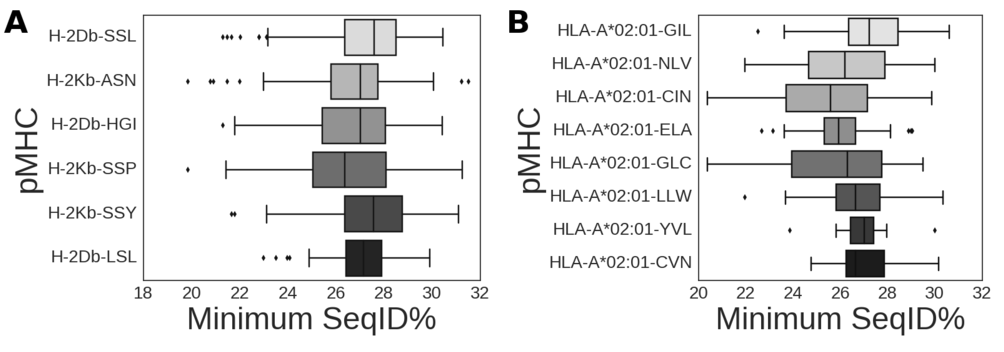

Supplement: Figure S1 — Minimum inner SeqID similarities between the TCRs that bind the same target. (A) Mouse alleles: H-2Kb, H-2Db. (B) Human allele HLA-A*02:01. [file Image_1.TIF]

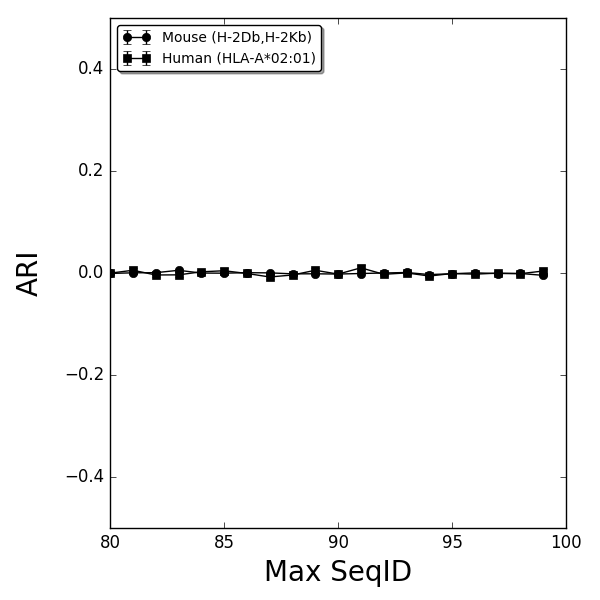

Supplement: Figure S2 — Prediction pipeline random performance. Random prediction is performed picking a random TCR when we search the database for the TopHit. Error bars are estimated using bootstrap with 1,000 iterations on the final prediction outcome. [file Image_2.TIF]

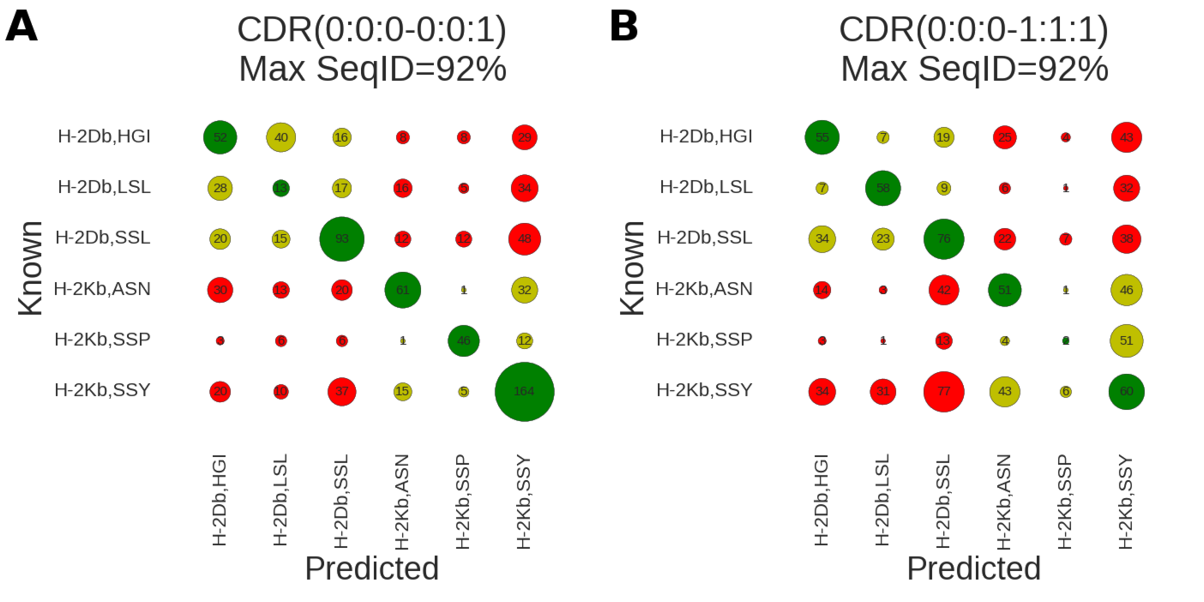

Supplement: Figure S3 — Contingency matrix for the prediction of mouse pMHC binders at a 92% Max SeqID threshold. (A) Prediction performed using only CDR3 β loop [model CDR(0:0:0–0:0:1)] with an Adjusted Rand Index (ARI) equal to 0.14. (B) Prediction performed using CDR1, CDR2, and CDR3 loops weighted equally [model CDR(0:0:0–1:1:1)] with an ARI = 0.04. [file Image_3.TIF]

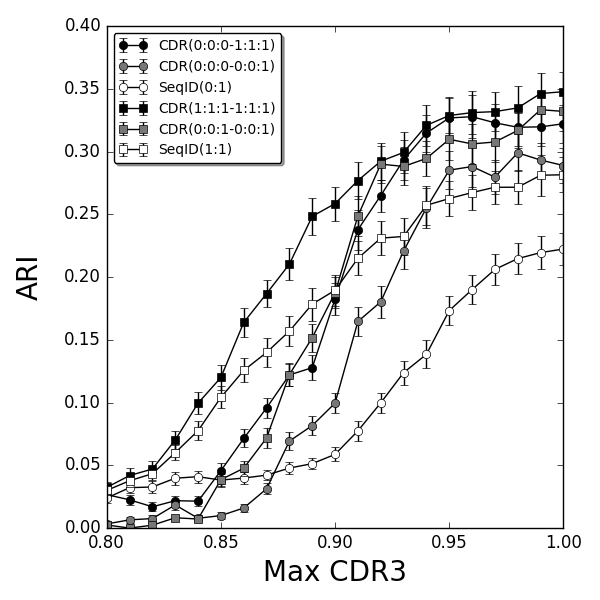

Supplement: Figure S4 — TCR prediction performance as a function of maximum CDR3 similarities. Using increasing CDR similarities from using only CDR3 β, CDR(0:0:0–0:0:1) to all α and β CDR loops, CDR(1:1:1–1:1:1). When we use only β chain we filter sequences using CDR(0:0:0–0:0:1) and when we predict using both chains we filter sequences using CDR(0:0:1–0:0:1). Error bars are estimated using bootstrap with 1,000 iterations on the final prediction outcome. [file Image_4.TIF]

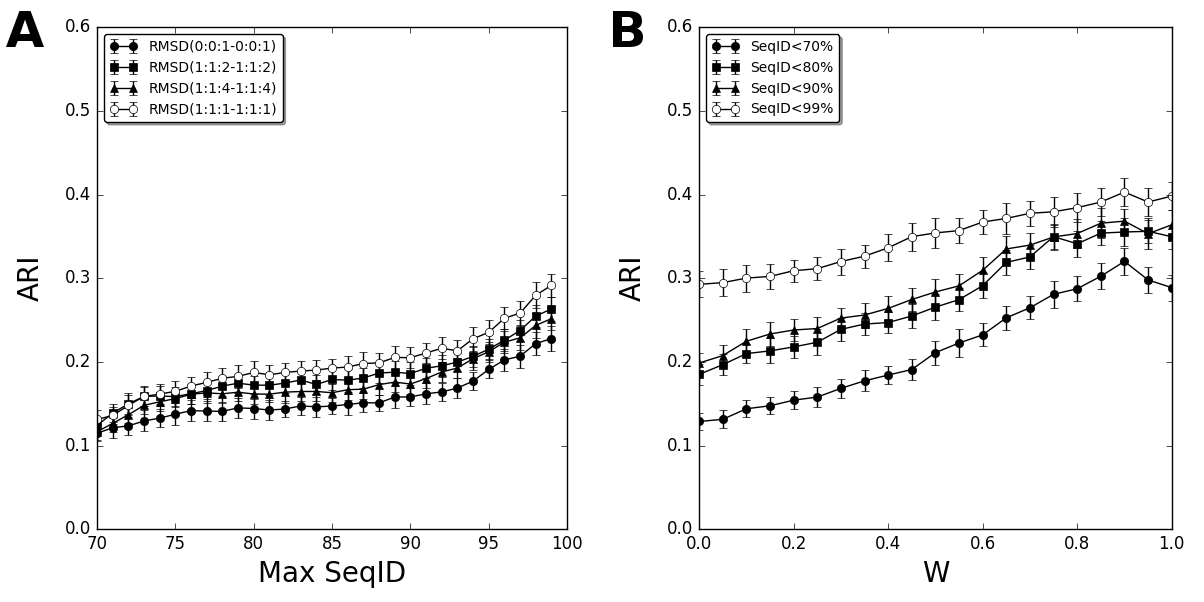

Supplement: Figure S5 — Looking for the best weights to combine structural similarity using the mouse benchmark. (A) RMSD prediction performance as a function of maximum SeqID allowed in the database for different weights. (B) Grid search for combined model weight between sequence and structural similarities different SeqID% cutoffs. [file Image_5.TIF]

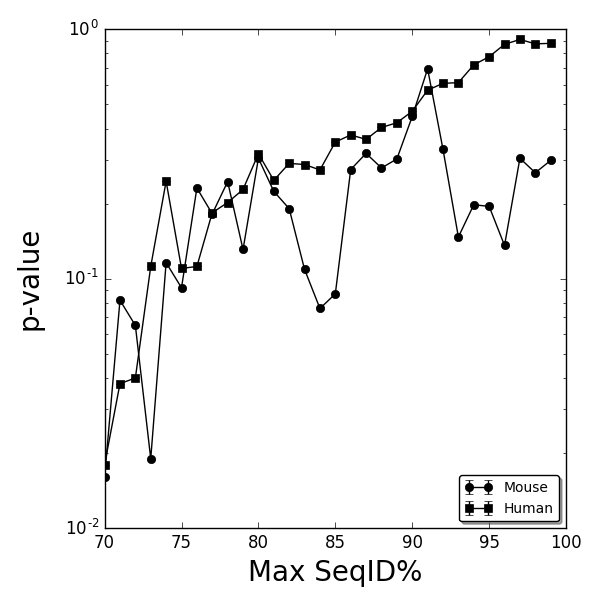

Supplement: Figure S6 — Bootstraping p-values comparing CDR+RMSD against CDR(1:1:4–1:1:4) as a function of maximum SeqID%. Tests were performed with 1000 iterations bootstrapping on the final prediction outcome for both models and p-value is obtained dividing by 1,000 the number of times the ARI value of CDR(1:1:4–1:1:4) resulted better than CDR+RMSD. [file Image_6.TIF]
