# Supplementary material for: T-Cell Receptor Cognate Target Prediction Based on Paired α and β Chain Sequence and Structural CDR Loop Similarities
Source: Front Immunol. 2019 Aug 28;10:2080. doi: 10.3389/fimmu.2019.02080 (PMC6724566; doi:10.3389/fimmu.2019.02080)
Supplement: Table S1 — Number of TCRs in datasets used for model discovery (mouse) and validation (human). [file Table_1.DOCX]

| **MHC** | **Peptide** | **#TCRs** | **Dataset** |
| --- | --- | --- | --- |
| HLA-A*02:01 | GILGFVFTL | 144 | Human |
| HLA-A*02:01 | LLWNGPMAV | 142 | Human |
| HLA-A*02:01 | NLVPMVATV | 78 | Human |
| HLA-A*02:01 | GLCTLVAML | 58 | Human |
| HLA-A*02:01 | CINGVCWTV | 53 | Human |
| HLA-A*02:01 | ELAGIGILTV | 27 | Human |
| HLA-A*02:01 | YVLDHLIVV | 10 | Human |
| HLA-A*02:01 | CVNGSCFTV | 8 | Human |
| H-2Kb | SSYRRPVGI | 251 | Mouse |
| H-2Db | SSLENFRAYV | 200 | Mouse |
| H-2Kb | ASNENMETM | 157 | Mouse |
| H-2Db | HGIRNASFI | 153 | Mouse |
| H-2Db | LSLRNPILV | 113 | Mouse |
| H-2Kb | SSPPMFRV | 74 | Mouse |

**Table S1. Number of TCRs in datasets used for model discovery (mouse) and validation (human).**
